# Emotional experiences in surrogate mothers: A qualitative study

**Published:** 2014-07

**Authors:** Hoda Ahmari Tehran, Shohreh Tashi, Nahid Mehran, Narges Eskandari, Tahmineh Dadkhah Tehrani

**Affiliations:** 1 *Research Center for Medicine and Religion, Qom University of Medical Sciences, Qom, Iran.*; 2 *Fertility and Infertility Research Center, Isfahan University of Medical Sciences, Isfahan, Iran.*; 3 *Faculty of Nursing and Midwifery, Qom University of Medical Sciences, Qom, Iran.*; 4 *Faculty of Nursing and Midwifery, Shahid Beheshti University of Medical Science, Tehran, Iran*

**Keywords:** *Surrogate mothers*, *Emotion*, *Experiences*, *Qualitative study*

## Abstract

**Background:** Surrogacy is one of the new techniques of assisted reproduction technology in which a woman carries and bears a child for another woman. In Iran, many Shia clerics and jurists considered it permissible so there is no religious prohibition for it. In addition to the risk of physical complications for complete surrogate mothers, the possibility of psychological complications resulted from emotional attachment to a living creature in the surrogate mother as another injury requires counseling and assessment prior to acceptance by infertile couples and complete surrogate mothers.

**Objective:** The purpose of this study was to assess the emotional experiences of surrogate mothers.

**Materials and Methods: **This was a qualitative, phenomenological study. We selected eight complete surrogate mothers in Isfahan. We used convenient sampling method and in-depth interview to collect the information. The data analysis was fulfilled via Colaizzi’s seven-stage method. Reliability and validity study of the roots in the four-axis was done.

**Results: **The findings of these interviews were classified into two main themes and four sub themes: acquired experiences in pregnancy (feelings toward pregnancy, relationship with family, relatives and commissioning couple) and consequences of surrogacy (complications of pregnancy, religious and financial problems of surrogacy).

**Conclusion:** Surrogacy pregnancy should be considered as high-risk emotional experience because many of surrogate mothers may face negative experiences. Therefore, it is recommended that surrogates should receive professional counseling prior to, during and following pregnancy.

## Introduction

Surrogacy is one of the new techniques of assisted reproduction technology in which a woman bears a child for another woman. The first successful pregnancy with uterine rent was reported 26 years ago; in 1985 (1). Surrogacy has been a controversial technique among assisted reproductive technology (ART) in recent years (2). It is illegal in countries such as Germany and Sweden also there are specific laws in countries such as France, the Netherlands, Denmark, Australia and some states of USA (3). This method, as an option for many infertile couples to have children has attracted the interest of many couples in Iran (4). According to Shia clerics and jurists, in-vitro fertilization (IVF) is permitted if noun righteous and objectionable actions such as masturbation, religiously forbidden touch /look occur in process of artificial fertilization (5).

There are two arrangements of Surrogacy: complete (gestational) and partial (genetic or traditional) (6). Gestational surrogacy necessarily involves IVF because the ovule does not belong to surrogate mother. The embryo is a combination of the intending parent/s gametes and/or donated gametes/embryos which are then transferred to the surrogate mother’s uterus. Therefore, there are variable possibilities for genetic relationships between the baby and the intending parents in a surrogacy arrangement. Gestational surrogacy is preferable and more common arrangement now (3, 7). 

The use of ART may have emotional effect on host woman or ovule-donor mother for example it can be guessed that giving back the child to the intending couple after birth may be a cause of emotional distress in rented mother (8-10). Important biological bonds are established between mother and her fetus during pregnancy. One of the most concrete examples of the importance of this bond comes from knowledge of fetal-maternal physiology. Oxytocin hormone plays a crucial role in priming the pregnant mother to respond in accordance with her natural maternal instincts (11). 

Therefore, there is a concern that the rented mother establishes such a firm emotional bonding to the fetus that separation of the newborn after giving to commissioning couple may be very difficult because she believes that the baby belongs to her and not to the commissioning couple. There is a risk of postpartum depression and psychologically harmful feeling of guilt or anger in these women. Some women undergo surrogacy because of economic problems without full awareness of the potential risks. It is also possible for the rented mother to be rejected by neighbors and friends that have deleterious effects on her psychological health as well her family's (7, 10). There have been very few studies on social and psychological aspects of surrogacy (9, 12, 13).

Therefore, these aspects of surrogacy are still the most controversial and challenging issues in reproductive processes in most countries. Although, this method has many advantages but has raised a lot of social- ethical questions and issues that requires comprehensive assessment in all its positive and negative aspects (12, 14). Review of literature shows that researchers have not paid serious attention to emotional experiences in surrogate mothers in limited researches conducted in this field. Phenomenology is an efficient approach for profound research on personal meanings, live experiences and a deep understanding of a phenomenon including communications, expectations, attitudes and beliefs (15). 

Diversity, similarities and differences in surrogacy can be explained by a phenomenological approach based upon the surrogate's perception. There is a comprehensive study on different aspects of surrogacy, gamete/fetus donation (legal, ethical, juridical). Emotional experiences of complete surrogate mothers can be significantly influenced by ethnical, cultural and national factors but we could not find any well-organized data or expanded and specific studies on these domains in Iran (4). Furthermore, knowledge and recognition about theses emotional experiences can help to find suitable solutions for its problems. Considering three above mentioned points, this phenomenological study aimed at exploring emotional experiences in Iranian complete surrogate mothers.

## Materials and methods

According to Parahoo, the design selected for a research should be the most suited one to achieve an answer to the raised question (16). The researcher designed and conducted a phenomenology qualitative research to explore surrogate mother's experiences. Qualitative research is a systematic subjective approach to describe life experiences that have not been investigated before and give them meaning (15, 17). Qualitative study provides the opportunity to explore behaviors, perspectives, feelings, and experiences in depth as well as quality and complexity of a situation through a holistic framework (18). Qualitative approach is proper to investigate new areas and topics which little is known about them and allow researcher to explore participants’ experience that have not investigated before (17). Qualitative approach has the ability to explore the various aspects of complex behaviors, attitudes and interactions that quantitative methods cannot (18, 19). The present qualitative study with a naturalistic paradigm tries to describe human experience in the circumstances that they have been gained because many phenomena in health-care are not quantitatively measurable (20). The current study was approved by the ethical committee of Qom University of Medical Sciences and was conducted from January to July 2012 in Isfahan, Iran.

The participants included 8 uterus-donor women who referred to Fertility and Infertility Center of Isfahan. The interviews were conducted after delivery. Inclusion criteria were: ability to express their experiences and having complete surrogate pregnancy. Sampling was purpose based and continued up to 8 samples that data saturation or repetition of previously collected data occurred. All participants were selected based on a common experience (surrogacy). At the onset of research, objectives of the study were explained to participants and they were assured of privacy of information. 

As previously stated, the researcher intended to carry out a qualitative study so it would require one round open ended, semi-structured, in-depth interviews. The researcher chose open-ended interviews as it allowed participants to discuss their opinions, feelings and experience fully in detail whereas a set interview with closed ended questions may inhibit them to express their full opinions and feelings. Using a semi-structured interview, the researcher prepared a topic guide or a series of determined questions to be answered by each participant (19). An individual face to face interview not only allows the researcher to observe any non-verbal communication but also allows both the interviewer and interviewee to seek any necessary clarification. The time of interview was unlimited and depended on interview process and lasted approximately 45 minutes (35 - 55 min). In addition, Socio-demographic data was recorded solely to describe the participants better. The questions were designed based on the contents of interview guide. The sequence of questions was different for each participant and depended on the circumstance of interview and given answers.

Participant's response was the guide for next questions. However; interview guide ensured that the researcher would ask the participants all questions required (17). Question-guide included three questions that two more questions were added based on answers of participants after the first and second interviews. We had no filled note.

**Interview-forms contained two categories of questions:**

1- Basic interview questions

2- Follow-up questions

Each interview session ended with two questions: "In your opinion, are there any questions that I would have posed to you?" and "Do you have any questions?" Data collection continued until data saturation when researcher could not obtain new findings. The researcher explained to participant that she could also talk about her thoughts, feelings, perceptions, and expectations associated with surrogacy and her experience that were not included in the framework of questions. To encourage and respect the participants, the researcher permitted them to ask any questions about the medical issues of surrogacy to complete their information and also gave them a call number to be available after that interview. Each interview was recorded, transcribed and analyzed to provide necessary feedback for sufficiency and saturation of data. After transcription of the first interview, the contents were assessed in order to find out whether or not they were complete and comprehensive. 

The Colaizzi seven-step method was used to analysis the data. At the first step, the researcher listened to participant^'^s speech carefully several times then transcribed and wrote down all words and reviewed them several times in order to understand the participant^'^s meaning correctly and profoundly. In cases that speeches were ambiguous, the researcher contacted with the participants by telephone to check the meanings and clarify. At the second step, the researcher derived important words and sentences relevant to the issue from the context of interviews and constituted the concepts. The first and second steps performed after each interview. At the third step, after finishing all the interviews, the researcher formulated concepts into codes. At the forth steps, the codes were categorized into specific clusters of themes and themes.

At the fifth step, all themes merged for an exhaustive description and constitution of whole structure of the phenomenon "the emotional experiences of surrogate mother". At the sixth step, to describe the fundamental structure of the phenomenon obviously. Thereafter, the researcher sought an expert researcher who reviewed the findings in terms of richness and completeness to provide sufficient description and to confirm that the exhaustive description reflected the experience of surrogate mothers. Finally, the seventh step aimed to validate study findings using "member checking" technique. It was performed through returning the research findings to the participants and discussing the results with them. Participants' views on the results of the study were obtained directly. Eventually, all participants approved that the results reflected their feelings and experience entirely and accurately.

The constant presence of the question: "what constitutes the essence of surrogate mothers experience?" in the whole process of the study led to extraction of themes and their interpretations. In this study, distinguishing the main themes allowed the researcher to expand the narrative description of emotional experience of surrogate mothers. In a phenomenology study, the researcher is not going to test a hypothesis but to extract the concepts of a phenomenon through clarification of related and involved individuals by referring them to their experience. To determine the validity and reliability of findings of present study the criteria of Lincoln and Guba were used: Credibility, transferability, dependability, and conformability (21). To improve the validity of findings in this study peer debriefing (external checking) and member checking was accomplished. The researchers were engaged in the study for a long time, around 10 months, and immersed themselves in the data. Transferability shows whether findings are utilizable and applicable to other settings and groups or not. We tried to introduce and explain the process of our study in detail and precisely so that other researchers could repeat it. To achieve dependability and conformability, in addition to in detail explanation of the process of study, inquiry audit was applied. 

**Statistical analysis**

To analysis the data, the Colaizzi seven-step method was used. The Colaizzi method of phenomenology uses Husserlian phenomenology to describe the essential structure of a phenomenon in its analysis. It was used to investigate the real-life experience of nurses who gave spiritual care (21, 22). This data analysis method appeared to be an appropriate methodology for the present study because it focused on finding the essence and meaning of the experiences of surrogate mothers (23). Prior to describing the analytical procedure of dataset, a brief description of data collection and transcripts formation are summarized as follow: 

Semi-structured, face-to-face interviews were conducted using a pre-prepared interview guide. The participants were encouraged to talk freely and tell stories using their own words. At the end of each interview, the researcher mentioned to the participants that another contact via telephone call would be required to discuss the findings of study in order to make sure that the findings reflected their own experience accurately. Data saturation was assessed parallel to data collection simultaneously by the main researcher and also another expert researcher in qualitative study. Then, saturation was confirmed based on consensus between both researchers. Eventually, the transcripts were double-checked by the same researchers mentioned above. The Analysis of the sixth interview did not yield any new codes so saturation had been achieved however two more interviews were performed and sampling was completed with 8 interviews. 

The following steps represent the Colaizzi process for phenomenological data analysis. First each transcript should be read and re-read repeatedly in order to obtain a general sense about the whole content. At second step the significant statements related to the phenomenon under study should be extracted from each transcript. These statements must be recorded on separate sheets noting their pages and line numbers then the concepts should be formulated from these significant statements. Following the formulation, the concepts (codes) should be sorted into the clusters of themes, and the themes. Then the findings of the study should be integrated to constitute an exhaustive description of the phenomenon under study and the fundamental structure of the phenomenon should be described. 

Finally, the validity of the findings should be assessed through comparing the researcher's descriptive results with participants' experiences (24).

## Results

Eight women aged 29-34 years old were included the study. All participants had children (1-4) and their educational levels varied from elementary to high school diploma (Table I). Findings of this qualitative study were classified into two main themes and four sub themes: the acquired experiences in pregnancy (with two sub-themes the feelings toward pregnancy), the relationship with family, relatives and the consequences of surrogacy (the complications of pregnancy, the religious and financial problems of surrogacy). 

In this study, one of the main contents as experiences acquired in pregnancy was emerged. There were a lot of important various points in participants' statements about emotional issues related to surrogacy. But the tips that most of them pointed out included the following (Table II).

**Experiences acquired in pregnancy**

**1. Feelings toward pregnancy**

**Coercion** **to have no feeling to baby:**

All participants stated that they tried to have no motherhood feeling to the child inside their womb. For instance, a participant said: *"That baby would never belong to me. I only provided an appropriate environment for the baby in my womb to be born and delivered to his/her parents. That was the easiest type of a child nursing".*

**Fear and worry about being baby abnormal/baby health** **:**

Fear and concern about the baby's abnormality was one of the unpleasant and annoying emotional experiences of uterus donors. For example, Zahra, one of the uterus donors, said: *“I was always worried that this child would be retarded. My sister said that "don’t worry because your child is health" but, actually that was not my own child. That was child of someone else. I thought if the baby was abnormal, maybe his/her commissioning couple didn’t want him/her. Thereafter what could I do with a retarded baby.”*

**2. Relationship with family, relatives and the main parents of fetus**

**Fear of husbands reactions in marital relationship** **:**

The fear of husband's reactions in marital relationship is one of the emotional experiences in uterus donors. As, one of the donors stated about her marital relationship: *“The sexual relationship between my husband and I was in trouble. He didn't tell me anything, but I figured out that he wasn't willing to have intercourse with me because he thought that somebody else's baby was in my belly. I got very upset but I tried not to bug shim”.*

**Doubt about informing her own children of the pregnancy type** **:**

One participant stated: *“I have a little girl who is very smart and understands many things so I did not know how to tell her. She frequently asked: "Mom, do you want to bring me a brother or a sister?”. I could not really explain it to her. I did not know what to say.”*

**Worries and concerns about informing the relatives and friends** **:**

The perception of the family members, relatives and friends of the surrogacy volunteers and also their responses about the issue of surrogacy were different. One participant stated that: *“None of my family members and relatives did know that I had rented my uterus except my mother and sister. I was very worried. I did not know if my mother-in-law found out, how she would react. I had to undergo this action because my husband was in a bad financial situation but I did not know what should I say to others?”*

Another participant said: *“My husband and I did not want another child because we had financial problems. I did not know how to tell the others I was pregnant while we had money issues. My husband said: “tell them it was an accident”. I was always worried that if other people found out I got pregnant this way, what would they think about us? ” *

**Consequences of surrogacy**

**3. The complications of pregnancy**

Hospitalization due to threatened abortion Maryam, 29 years old participants said: *"I was engaged in a terrible situation. In addition to emotional problems, I faced a medical problem too. I was hospitalized due to bleeding in early weeks. I was worry about my health. In addition, excessive worry of commissioning couple annoyed me too. Furthermore; I was concerned about my money because if the baby had been aborted I could not have received the agreed money".*

**4. The Religious and financial problems of surrogacy**

**Having no obvious religious legitimation and social acceptability** **:**

The fifth participant said: *"At first my husband and I did not like anybody to get to know I was pregnant because we were not sure about its religious righteousness. After referring to Shia scholars and jurists and asking our questions, we were partially assured. Indeed, I was not completely sure that surrogacy was religiously right although they said that the baby was theirs and did not belong to me and I was just going to keep it. Anyway, I had to do that because my husband was jobless for a while****".***

**No enough payment for expenses by the main parents** **:**

One of the participants named Hadith said: *“It was very difficult for me to ask somebody else for money. My husband did not give me any money during the nine months of pregnancy because he believed that the baby was not his but belonged to somebody else so the main father had to pay for everything but It was really hard for me to frequently ask for money." She went on saying at the end: "I wish the financial problems were resolved”.*

**Table I:** Socio-demographic information of surrogate mother

**Parameter**		**No. of cases (Mean±SD)**
Age (years)		31.6 ± 1.4
Having her own child		
	Yes	7
	No	1
Marital status		
	Married	7
	Widow	1
	Divorced	0
Occupation		
	Housewife	1
	Having a Part-time job	4
	Having a Full-time job	3
Type of surrogacy		
	Partial (genetic)	0
	Complete (non-genetic)	8
History of previous surrogacy		
	No	7
	Yes	1

**Table II:** The main themes and sub- themes in this study

**The main themes**	**The sub-themes**	**The code**s
Experiences acquired in pregnancy		
	Feelings toward pregnancy	
		Coercion to have no feeling to baby
		Fear and worry about being baby abnormal/baby health
	Relationship with family, relatives and the commissioning couples	
		Fear of husbands reactions in marital relationship
		Doubt about informing her own children of the pregnancy type
		Worries and concerns about informing the relatives and friends
Consequences of surrogacy		
	The complications of pregnancy	
		Hospitalization due to threatened abortion and elevated blood sugar
	The religious and financial problems of surrogacy	
		having no obvious religious legitimation and social acceptability
		No enough payment for expenses by the commissioning couples

## Discussion

The purpose of this study was to assess the emotional experiences of surrogate mothers. The findings of this qualitative study were classified into two main themes and four sub-themes. The first theme was "the acquired experiences in pregnancy" that included two sub-themes: the feelings toward pregnancy and the relationship with family and relatives. The second theme was "the consequences of surrogacy" that included two sub-themes: the complications of pregnancy and the religious and financial problems of surrogacy.

Coercion to have no fooling to baby is the first code of the first sub-theme (feelings toward pregnancy) of the first main theme (experiences acquired in pregnancy). It seems that the separation from the newborn and handing the child over to the commissioning couple will be a distressing and painful experience for a surrogate mother. However; there is inconsistent and conflicting evidence about the emotional effects of uterus donation process on surrogate mothers. For example, in a study by Ciccarelli, fourteen surrogate mothers were asked to report their feelings or concerns about relinquishing the child. One mother reported emotional distress over the relinquishment and two others reported a strong instinctual urge to bond with the child. The remaining eleven did not feel bonded with the child, which may seem to indicate that for the majority of surrogates the issue of having to relinquish the child did not appear to be a problem (25). Ber concluded that pregnancy can be painful for surrogate mothers as much as infertile mothers (26).

Some evidence shows that baby transfer may lead to considerable distress and emotional problems in uterus of donor mothers. On the other hand, there is a concern that lack of maternal attachment to the baby during the surrogacy process may be challenging for the health of both the mother and the baby (7). The important bond between mother and child, which derives from both biological and cognitive/psychological aspects of human nature, begins during pregnancy and continues after birth. Surrogacy ruptures this significant bond (10, 25). The study accomplished in England by Jadva showed that all of the surrogate mothers in postpartum period, with no doubt, delivered the babies according to previous agreement. The follow up of those women showed that 32% of women had emotional and psychological problems for several weeks after losing the babies. After a few months, this rate decreased to 15% and continued until 1 year only in 6% of cases (27). 

However; the rate of postpartum depression in surrogate women is not higher than the general population (2, 9). Also, most of researches don’t report serious psychological problems for embryo host mothers (8, 28, 29). In contrast, some studies indicate negative effects and psychological problems following surrogacy (6). In general, the results of the studies show that despite some worries about host mother's emotional problems, these problems do not threaten their psychological health (7). However; it is recommended that more attention should be paid to choosing suitable hosting applicants with professional counseling before pregnancy. 

In the current study, self-obligation to have no feeling to the child was the mothers' reaction to confront with this issue in 6 participants. The results of the Jadva’s study in 2003 showed that none of the surrogate mothers had any special problems after delivering the babies to the commissioning couples (27). This finding is similar to ours. Fear and worry about being baby abnormal/baby health as the second code of the first sub-theme (feelings toward pregnancy) of the first main theme (experiences acquired in pregnancy) is one of our findings. This finding has been reported in a few studies before (25).

Fear of husband reactions in marital relationship is the first code of the second sub-theme (relationship with family, relatives and the commissioning couples) of the first main theme (experiences acquired in pregnancy). In the present study most of the women were concerned about their sexual relationships during the pregnancy and eventually disruption of family relationship. There were a few surrogate mothers that complained about an insignificant decrease in libido. Jadva in a study on marital satisfaction of women found that 16% of the surrogate mothers had low marital satisfaction, 4% had severe problems in marital relationship with their spouse, and 80% had moderate or high marital satisfaction (27). So we can say that the surrogacy phenomenon have no significantly negative impact on the marital relationships of couples. This difference may be related to cultural or religious differences between the Iranian and England communities.

Doubt about informing her own children of the pregnancy type as the second code of the second sub-theme (relationship with family, relatives and the commissioning couples) of the first main theme (experiences acquired in pregnancy), was one of the concerns expressed by mothers participating in this study. According to the recommendation of the Medical Council of the UK, if surrogate mothers have children, they should talk to them about this issue because the absence of the baby in the family after birth could be a cause of conflict in their children (10). The results of Women's study showed that 52% of uterus donor mothers by complete arrangement preferred to tell the truth about the kind of pregnancy to their children, while this rate decreased to 24% in genetic surrogacy (30).

Worries and concerns about informing the relatives and friends is the third code of the second sub-theme (relationship with family, relatives and the commissioning couples) of the first main theme (experiences acquired in pregnancy). One of the significant issues affecting the emotional health of surrogate mothers is the attitude of friends, colleagues, friends, and general public towards surrogacy. Lack of social support due to negative attitude of people can make surrogate mothers psychologically vulnerable (6, 7).

Surrogate mothers maybe encounter annoying reactions of people. Jadva *et al* in a study on the experience of surrogate mothers in raising the issue to their families showed that in 7% of the cases, the family reaction was negative, in 48% was positive, and in 46% was neutral or mixed of negative and positive. The follow up study showed that family feeling toward this issue was positive in 76% of cases and only 3% of the cases had a negative feeling one year after child delivery (8). Also, Blise believes that there is a risk that surrogate mothers be humiliated by their families or friends that affect the psychological health of surrogate mothers (27).

Another study in Greece showed negative attitude of most people towards this method (10). Shenfield believes that people attitude is mainly resulted from unawareness about various aspects of the subject that can be solved somewhat by giving appropriate information and awareness. They found that sometimes some of families, family members and friends initially had a negative attitude to surrogacy but later accepted it and took pride in the host-mothers. The most of host-mothers reported their husband's support and their children's positive reaction. None of them had faced a serious trouble (8). The results of current study show that despite positive attitude of surrogate mothers, they believe that there are not appropriate cultural circumstances in the community. This caused them to resolve the problem with solutions like attribution the pregnancies to their husband, announcement of neonate death to others, declaring that the pregnancy is unwanted; stopping relationships with relatives and friends until delivering the baby to the commissioning couple. 

Some of them, according to their cultural circumstances made others aware of their decision. Husbands' awareness and consent, agreement of religious scholars with this method of pregnancy, religious legitimacy of embryos growing in their wombs and the altruistic aspect of surrogacy that helps to solve the problem of infertile couples were important factors that influence significantly on making decision of surrogate mothers and their husbands to inform others about the real cause of pregnancy. 

Hospitalization due to threatened abortion and elevated blood sugar as the first code of the first sub-theme (the complications of pregnancy) of the second main theme (consequences of surrogacy) occurred in two cases. Although pregnancy is a natural process, it may induce some risks. When a woman decides to become pregnant, she is prepared to face the risks. In surrogacy, unfortunately, she bears the risks without the natural benefit of motherhood. There is therefore a sense of futility if something goes wrong for her, which is especially the case if a woman becomes a surrogate merely for altruistic purposes. For example, the Daily Mail reported that a surrogate mother, aged 29 had died 90 minutes after giving birth due to aorta rupture following high blood pressure. Her mother Marilyn said:" Surrogacy caused Natasha's death. People must realize that childbirth isn't something you enter into lightly. It's still dangerous. 

Natasha didn't want any more children herself but she comes from a big family and she felt for people who couldn't have children*. *Her children had brought her a lot of pleasure so she wanted other parents to share some of that joy" (28). The literature regarding the medical risks associated with surrogate pregnancy is limited to a few case series. It remains to be determined if the obstetric risks are the same as those for any other pregnancy derived by in vitro fertilization with the same number of fetuses. Most case series report no increase in adverse events related to surrogate pregnancy; however, in a recent report, 2 of 9 surrogate mothers underwent postpartum hysterectomy: after giving birth to triplets with placenta accreta and after uterine rupture that occurred during delivery of a macrosomic infant (2, 29, 31).

A prenatal diagnosis of disability or perceived imperfection in surrogate mother could result in serious trouble with a surrogacy arrangement such as couple reneging. At least one such case has occurred in the US. In such cases, depending on the circumstances and severity, the option of abortion could be considered by the surrogate; however, differing moral perspectives on abortion have the potential to result in an irresolvable stalemate. The surrogate may still wish to proceed with the birth; however, the commissioning couple may no longer want the child. Alternatively, the surrogate may choose an abortion contrary to the wishes of the commissioning couple, but presumably the surrogate’s decision for abortion under law would prevail (32). 

We did not have any cases like this among the participants of the current study. Having no obvious religious legitimation and social acceptability is the second code of the second sub-theme (religious and financial problems of surrogacy) of the second main theme (consequences of surrogacy). Doubt about religious legitimacy was an unresolved problem. As an achieved themes of the present study that has not been reported in the related articles. Considering the fact that complete surrogacy is permitted by most of the Shia clerics and jurists, this problem can be somewhat resolved by improving public awareness. No enough payment for expenses by the commissioning couples is the third code of the second sub-theme (religious and financial problems of surrogacy) of the second main theme (consequences of surrogacy). 

In our study, lack of a well-written documented contract between the surrogates and commissioning couples caused surrogate mothers to face some problems including financial problems during pregnancy and be worried about receiving the agreed payment prior to pregnancy in cases the baby probably would be lost or have abnormalities. Abbasi believes it is crucial for both commissioning couples and the surrogates to have a legal contract to define obviously the responsibilities and rights of both sides and guarantee the issues under the contract such as the payment of money so that the surrogate mothers could spend their pregnancy peacefully (13). 

**Limitation**

The only limitation of the present study was that in Iran, genetic surrogacy arrangement is forbidden, so the sampling was limited to complete arrangement. 

## Conclusion

Our study showed mainly the positive experience of surrogate mothers however, surrogacy needs special care in various aspects that its management is very important and requires deep consultation to select suitable host. Meanwhile, counseling should be continued for surrogate mothers during and after pregnancy.
